# Expression of the beta-2 adrenergic receptor (ADRB-2) in human and monkey ovarian follicles: a marker of growing follicles?

**DOI:** 10.1186/s13048-015-0136-4

**Published:** 2015-03-07

**Authors:** Christoph Merz, Sabine Saller, Lars Kunz, Jing Xu, Richard R Yeoman, Alison Y Ting, Maralee S Lawson, Richard L Stouffer, Jon D Hennebold, Francis Pau, Gregory A Dissen, Sergio R Ojeda, Mary B Zelinski, Artur Mayerhofer

**Affiliations:** Anatomy III, Cell Biology, Ludwig-Maximilian-University Munich (LMU), Munich, Germany; Division of Neuroscience, Department of Biology II, LMU Munich, Munich, Germany; Division of Reproductive and Developmental Sciences, Oregon National Primate Research Center, OHSU, Beaverton, Oregon USA; Division of Neuroscience, Oregon National Primate Research Center, OHSU, Beaverton, Oregon USA

**Keywords:** Granulosa cell, Catecholamines, Growth, 3D Culture, Sympathetic Nervous System

## Abstract

**Background:**

ADRB-2 was implicated in rodent ovarian functions, including initial follicular growth. In contrast, ADRB-2 expression and function in nonhuman primate and human ovary were not fully known but innervation and significant levels of norepinephrine (NE), which is a ligand at the ADRB-2, were reported in the ovary.

**Methods:**

We studied expression of ADRB-2 in human and rhesus monkey ovary (RT-PCR, immunohistochemistry; laser micro dissection) and measured levels of norepinephrine (NE; ELISA) in monkey follicular fluid (FF). 3D cultures of monkey follicles (4 animals) were exposed to NE or the ADRB-2 agonist isoproterenol (ISO), and follicular development (size) was monitored. Upon termination expression of ADRB-2, FSH receptor and aromatase genes were examined.

**Results:**

Immunohistochemistry and RT-PCR of either human follicular granulosa cells (GCs) obtained by laser micro dissection or isolated monkey follicles revealed ADRB-2 in GCs of primordial, primary, secondary and tertiary follicles. Staining of GCs in primordial and primary follicles was intense. In large preantral and antral follicles the staining was heterogeneous, with positive and negative GCs present but GCs lining the antrum of large follicles were generally strongly immunopositive. Theca, interstitial, and ovarian surface epithelial cells were also positive. NE was detected in FF of preovulatory antral monkey follicles (0.37 + 0.05 ng/ml; n = 7; ELISA) but not in serum. We examined preantral follicles ranging from 152 to 366 μm in diameter in a 3D culture in media supplemented with follicle stimulating hormone (FSH). Under these conditions, neither NE, nor ISO, influenced growth rate in a period lasting up to one month. Upon termination of the cultures, all surviving follicles expressed aromatase and FSH receptors, but only about half of them also co-expressed ADRB-2. The ADRB-2 expression was not correlated with the treatment but was positively correlated with the follicular size at the beginning and at the end of the culture period. Hence, expression of ADRB-2 was found in the largest and fastest-in vitro growing follicles.

**Conclusions:**

The results imply ADRB-2-mediated actions in the development of primate follicles. Drugs interfering with ADRB-2 are used to treat medical conditions and may have unexplored effects in the human ovary.

## Background

The catecholamines norepinephrine (NE) and dopamine were found in high concentrations in human ovarian homogenates and in follicular fluid (FF) of preovulatory follicles. The source of these neurotransmitters is likely the documented innervation of the human ovary by the sympathetic nervous system or intra-ovarian neurons [1-7]. NE is the main neurotransmitter of the sympathetic nervous system and can affect receptor-bearing cells. However, potential targets and roles of NE in the human ovary are not well known.

Ovarian vascular cells and endocrine cells of the ovarian follicle of several species, including humans, possess different catecholaminergic receptors, including ADRB-2 [8-11]. Theca cells (TCs) and granulosa cells (GCs) express alpha- and beta-adrenergic receptors and their activation regulates, for example, the levels of cAMP and steroid production [7,12]. NE can also stimulate the initial, FSH-independent growth of small follicles in the neonatal rat in vitro [8]. Growth promoting actions initiated by ADRB-2 were described in other organs, for instance, in later stages of salivary gland growth [13] and in the differentiation of stem cells [14]. Thus ADRB-2 may have physiological roles in stimulating growth/differentiation of cells. This also appears to apply to tumor cells, but to our knowledge it is not known, whether ovarian tumors have been studied [15-18].

Recent data suggest that systemic exposure of rats to the ADRB-2 agonist isoproterenol in vivo affected follicular growth and androgen biosynthesis and induced a polycystic ovarian phenotype. These changes were prevented by propranolol, a blocker of ADRB [19]. Indeed derangements of the actions of NE via ADRB-2 may be related to the etiology of polycystic ovary syndrome (PCOS) in women. This condition causes anovulatory infertility and affects 5–10% of women of the reproductive age [20,21]. Evidence for hyper-innervation [22] of the human PCOS ovary was reported. Increased sympathetic activity associated with PCOS on one ovary, and the effectiveness of stress-reducing exercise and even acupuncture on the contralateral ovary within the same patient, support the notion of a crucial role of sympathetic innervation [22-26]. Experimental rat-PCOS models reveal an increased sympathetic tone and a dominant catecholaminergic ovarian innervation [24]. Sectioning of the superior ovarian nerve, which carries the majority of the sympathetic fibers of the rat ovary, increased ovarian ADRB-2 expression to normal levels, and restored estrous cyclicity and the ability to ovulate [2,27]. These data imply an important intra-ovarian role of NE, which is, at least in part, mediated via ADRB-2.

Although the (sympathetic) innervation of the ovary has been intensively studied [5,6], to date there are no studies on the precise localization of ADRB-2 in the human ovary. Furthermore, the possibility that ADRB-2 may regulate follicular growth in human or nonhuman primates has not been investigated. Yet, blockers of NE receptors of the beta-type, so called beta-blockers, are used for treating hypertension, angina, myocardial infarction, arrhythmias and heart failure. Some of them target both adrenergic receptors ADRB-1 and 2 [28] and hence knowledge about ovarian targets appears desirable. We addressed these issues using human and rhesus monkey ovarian sections and a 3D culture system of preantral monkey follicles.

## Methods

### Ovarian sections

The human and rhesus monkey ovarian samples used for immunohistochemistry were identical to the ones described in previous studies [29,30]. Additional sections were cut from the blocks and used as described below.

### Follicular culture

General care and housing of rhesus macaque monkeys (Macaca mulatta) were provided by the Division of Comparative Medicine at the Oregon National Primate Research Center (ONPRC). The studies were conducted in accordance with the NIH Guide for the care and Use of Laboratory Animals and all protocols were approved by the ONPRC Animal Care and Use Committee. Animals were pair caged in a temperature-controlled (22°C) light-regulated 12L:12D room. Diet consisted of Purina monkey chow (Ralston-Purina, Richmond, IN), provided twice a day, supplemented with fresh fruit or vegetables once a day and water ad libitum. Ovaries were collected from 4 adult female rhesus macaques during necropsy for other research or due to illness other than reproductive health issues. Ovaries from 3 additional animals were used for pilot studies in order to establish the culture conditions. The age of the animals ranged from 7–12 years. Blood samples were collected prior to the removal of the ovaries, for steroid hormone measurements. Ovaries were immediately transferred into HEPES-buffered holding media (CooperSurgical, Inc., Trumbull, CT, USA) supplemented with 0.2% (v/v) human serum protein supplement (SPS, CooperSurgical, Inc.) and 10 μg/ml gentamicin (Sigma-Aldrich, St Louis, MO, USA) at 37°C.

Ovarian tissue processing and 3D culture were described previously [31-33]. In brief, secondary follicles were dissected mechanically from cortical strips using 31-gauge needles. Only multilayered secondary follicles (containing at least three layers of GCs) that displayed the following characteristics were selected for encapsulation in alginate hydrogel: 1) no clear antral cavity, 2) an intact basement membrane with attached stroma, and 3) a visible oocyte that was round and centrally located within the follicle. Retrospective measurements showed that for our studies the follicle diameters ranged from 152 to 366 μm. Follicles were encapsulated in beads composed of 0.25% sodium alginate (w/vol; 55%–65% alginic acid; FMC BioPolymers (Philadelphia, PA) and randomly transferred to individual wells of a 48-well plate. For controls one group was cultured with media containing 300 μl of alpha minimum essential medium supplemented with 0.3% (v/v) human serum protein supplement (CooperSurgical, Inc.), 1 mg/ml bovine fetuin (Sigma-Aldrich, St. Louis, MO), 5 μg/ml insulin, 5 μg/ml transferrin, 5 ng/ml sodium selenite and FSH (3 ng/ml recombinant human (rh) FSH (NV Organon, Oss, Netherlands). In a second group 100 nM norepinephrine (NE; Sigma, St Louis, MO, USA) and in third group 100 nM of the non-selective ADRB-agonist isoproterenol (ISO; Sigma) was added. Incubation was carried out at 37°C in a 5% oxygen environment (in 6% CO_2_/89% N_2_) for approximately 30 days. Half of the culture media (150 μl) was exchanged every other day.

### Assessment of follicle survival and growth and RT-PCR

Follicle survival and diameters were assessed weekly [31] using an Olympus (Tokyo, Japan) CK40 inverted microscope attached to an Olympus PD11 digital camera (Olympus Imaging America Inc., Center Valley, PA, USA). Photographs of each follicle were collected and imported to ImageJ 1.45 software. Follicles were measured from the outer layer of cells at the widest diameter of the follicle and a second measurement perpendicular to the first. The mean of these two values was then calculated and named as the diameter of the follicle. Follicles were classified as degenerating if the oocyte was no longer surrounded by a layer of GCs, the oocyte was dark, the GCs had become dark and fragmented, or the diameter of the follicle decreased.

At the end of the culture period, all surviving follicles were harvested and frozen until extraction of RNA (Absolutely RNA Nanoprep Kit, Stratagene, Agilent Technologies Inc., Santa Clara, CA, USA). For control purposes, RNA was also extracted from freshly isolated follicles that were not cultured. RNA (200–500 ng) was subjected to reverse transcription, using random primers (pdN6) and SuperScript II Reverse Transcriptase, 200 U/μl (Invitrogen GmbH, Darmstadt, Germany). PCR steps consisted of 35 cycles of denaturing (at 94°C for 60 sec), annealing (at 60°C for 30 sec), and extension (at 72°C for 60 sec). Reaction tubes lacking the RT product input or RNA input instead of cDNA were used as PCR controls. The identities of all PCR products were verified by direct sequencing, using one of the specific primers (Table 1).

**Table 1:** **Oligodeoxynucleotide primer pairs used in (RT-) PCR experiments**

**Type**	**Primer sequence (5′–3′)**	**GenBank accession no.**
Aromatase		
Sense	gcc ttt ttc tct tgg tgt gg	NM_000103
Antisense	atc ccc atc cac agg aat ct	NM_000103
ADRB-2		
Sense	gag cac aaa gcc ctc aag ac	NM_000024
Antisense	tgg aag gca atc ctg aaa tc	NM_000024
Nested-sense	tag gca tca tca tgg gca	NM_000024
FSHR		
Sense	ctg ctc ctg gtc tct ttg ct	NM_000145.2
Antisense	ggt ccc caa atc ctg aaa at	NM_000145.2

### Laser micro dissection (LMD) and RT-PCR

Human ovaries embedded in paraffin (Anatomie III, Cell Biology; used in previous studies, e.g. [3,29,30]) were cut into 5 μm sections and mounted onto a 1.35-μm polyethylene naphthalene membrane pasted to a glass slide, which had been pretreated with UV light for 30 min. The sections were deparaffinized and processed for LMD as previously described [34]. A nitrogen laser of the Robot-MicroBeam (P.A.L.M. GmbH Mikrolaser Technologie, Bernried, Germany), was used to circumscribe and then isolate GCs of large follicles. Samples were ejected from the object slide and catapulted directly into the cap of a microfuge tube. 50 μl of RNA stabilization reagent (RNEasy Protect Mini-Kit, Qiagen, Hilden, Germany) were added into the cap. Samples were frozen at -70°C until RNA extraction (RNEasy, Qiagen). RT followed by two nested PCR amplifications was performed. The identities of all PCR products were verified by direct sequencing, using one of the specific primers (Table 1).

### Immunohistochemistry

Human (n = 4) and monkey (n = 6) ovarian sections were subjected to immunohistochemistry as described [3,34]. Two different specific antisera to human ADBR-2 were used: rabbit anti-ADBR2 at 1:50–1:100 dilution (Life Span BioSciences Inc., Seattle, USA), and rabbit anti-ADBR-2 at 1:400 dilution (IMGENEX Corporation, San Diego USA). Secondary antisera (anti-rabbit biotinylated gamma globulin; 1:250; Vector Laboratories, Inc. Burlingame, CA; USA), and avidin-biotin complex peroxidase (ABC, Vector Laboratories) were used. The reactions were visualized with diamino-benzidine (DAB; Sigma). For control purposes, the primary antisera were replaced by buffer, non-immune serum or rabbit IgG. Some sections were counterstained with hematoxylin in order to visualize cell nuclei. Sections were observed with a Zeiss Axiovert microscope (Zeiss, Jena, Germany).

### Controlled ovarian stimulation (*COS) protocol/NE ELISA*

Follicular fluid (FF) was obtained from additional regularly cycling female rhesus macaques undergoing COS protocols as previously described [35]. Samples for NE ELISA stem from animals, which did not receive an ovulatory human chorionic gonadotropin stimulus (1000 IU single injection i.m. (hCG, Novarel; Ferring Pharmaceuticals) to initiate periovulatory events (n =7). Individual preovulatory follicles (1–2 per ovary) were aspirated and pooled using a 22-gauge needle during laparoscopy from anaesthetized animals. The FF was centrifuged to separate out GCs and blood cells. Blood samples were obtained at the same time. Concentration of NE in FF and serum was analyzed in duplicate at the Endocrine Technology Core Lab (ONPRC) by a specific extraction-ELISA kit (Norepinephrine (Research) ELISA, Cat# 17-NORHU-E01-RES, ALPCO, Salem, NH). This kit assay was validated for monkey serum and FF. The assay has negligible cross-reactivity with at least 13 monoamines including epinephrine and dopamine. The sensitivity of the assay was 0.2 ng/ml, with a range of detection to 32 ng/ml. All FF samples were analyzed in one kit. The intra-assay variation was estimated at 8.5%.

### Statistical analysis

Data were analyzed using Prism 5 (GraphPad Software, San Diego, CA, USA). Results of ELISA measurements data were analyzed by repeated-measures ANOVA followed by the Newman-Keuls post-test. The data on follicle diameter and growth were analyzed applying nonparametric tests (see Results) since we have not assumed Gaussian distribution (see Results). Data were considered significant at P < 0.05 or less.

## Results

### ADRB-2 expression in human and monkey ovary

**Figure 1:**
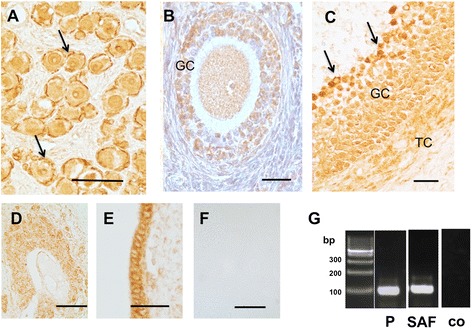
**ADRB-2 in monkey ovary.** Expression by granulosa cells (GC), thecal cells (TC) **(A-C)**, interstitial cells and surface epithelium **(D, E)** in representative sections. Control (IgG instead of antiserum) **(F)**. Bars: 30 μm. The section shown in B was counterstained to visualize the nuclei of immuno-positive and immuno-positive GC. Confirmation of ADRB-2 (100 bp) mRNA in isolated monkey follicles by semi-nested RT-PCR small (P: preantral follicles; SAF: small antral follicles; pools of 3 individual follicles) **(G)**; co: control with RNA instead of cDNA.

**Figure 2:**
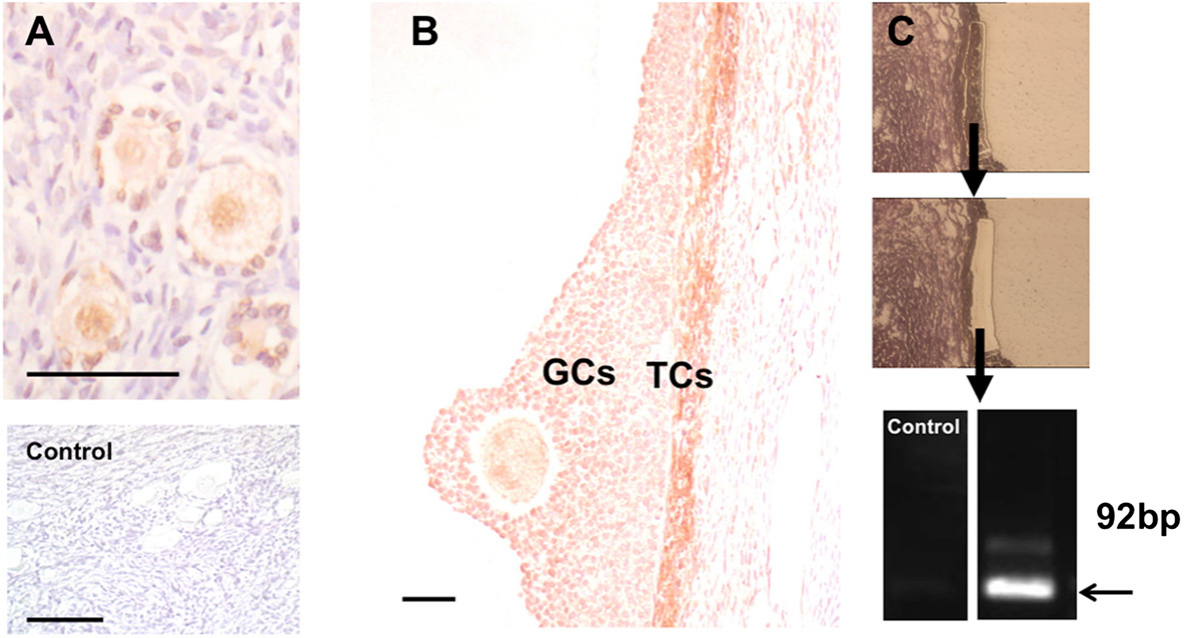
**ADRB-2 in human ovary.** ADRB-2 in small **(A)** and large antral **(B)** human follicles. Control (B, bottom). Bars: 50 μm. **(C)** Detection of ADRB-2 (100 bp) in GCs of large human follicles. Sections before (top) and after excision (middle) by LMD and RT-PCR result (bottom). Control: no input cDNA.

Immunohistochemistry identified ADRB-2 in GCs and in TCs of human and monkey follicles (Figures 1 and 2). Robust staining was observed in primordial and primary follicles (in postnatal and adult ovary (Figures 1A and 2A). Staining of GCs in growing preantral and small antral follicles was patchy and seen in most, but not all, GCs (Figure 1B). In large antral follicles, staining of GCs was in general heterogeneous, but the cells lining the antrum were stained strongly (Figures 1C and 2B). Other ovarian cells were immuno-positive, namely ovarian interstitial gland and surface epithelial cells (Figure 1D, E). Both antisera showed the same pattern (data not shown). All controls performed were negative (see Figures 1F and 2A). Expression of ADRB-2 by intact monkey preantral and small antral follicle was confirmed by RT-PCR followed by sequencing (Figure 1G). In addition, a semi-nested RT-PCR detected ADRB-2 in GCs of a large human follicle after excision by LMD (Figure 2C).

### NE in follicular fluid of preovulatory follicles of rhesus monkey

The presence of NE in FF and thus in the monkey follicular GC compartment, which lacks nerve fibers, was confirmed using samples from preovulatory follicles obtained from animals prior to receiving exogenous gonadotropins to induce the growth of multiple antral follicles (0.37 + 0.05 ng/ml; mean + SEM; n = 7; ELISA). As recently found in human FF [3], macaque FF NE levels exceeded those in serum, which were below the detection limit of 0.2 ng/ml in monkeys.

### Growth of monkey follicles in 3D culture

**Figure 3:**
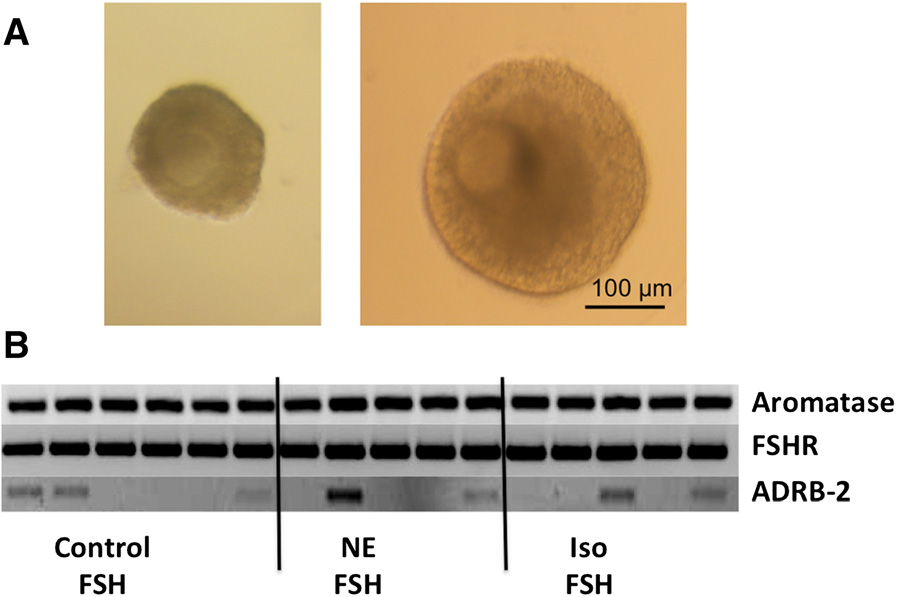
**Monkey secondary follicles grown in a 3D culture system**. Example of a monkey secondary follicle grown in a 3D culture system: Size at the beginning (left) and at the end of the culture when this follicle achieved the small antral stage (right; 35 days) **(A)**. All 16 follicles that were examined upon termination of the culture express aromatase and receptors for FSH (FSHR), but only a few (7) express ADRB-2 **(B)**.

Multilayered, secondary follicles grew in 3D culture and developed an antrum (Figure 3A). At the end of the approximately 1 month culture period, surviving follicles (for criteria see definition in materials and methods) expressed aromatase and FSHR mRNA. However, only a fraction of those also co-expressed ADBR-2 mRNA (Figure 3B). Neither ISO, nor NE treatment during culture affected ADBR-2, aromatase or FSHR mRNAs.

**Figure 4:**
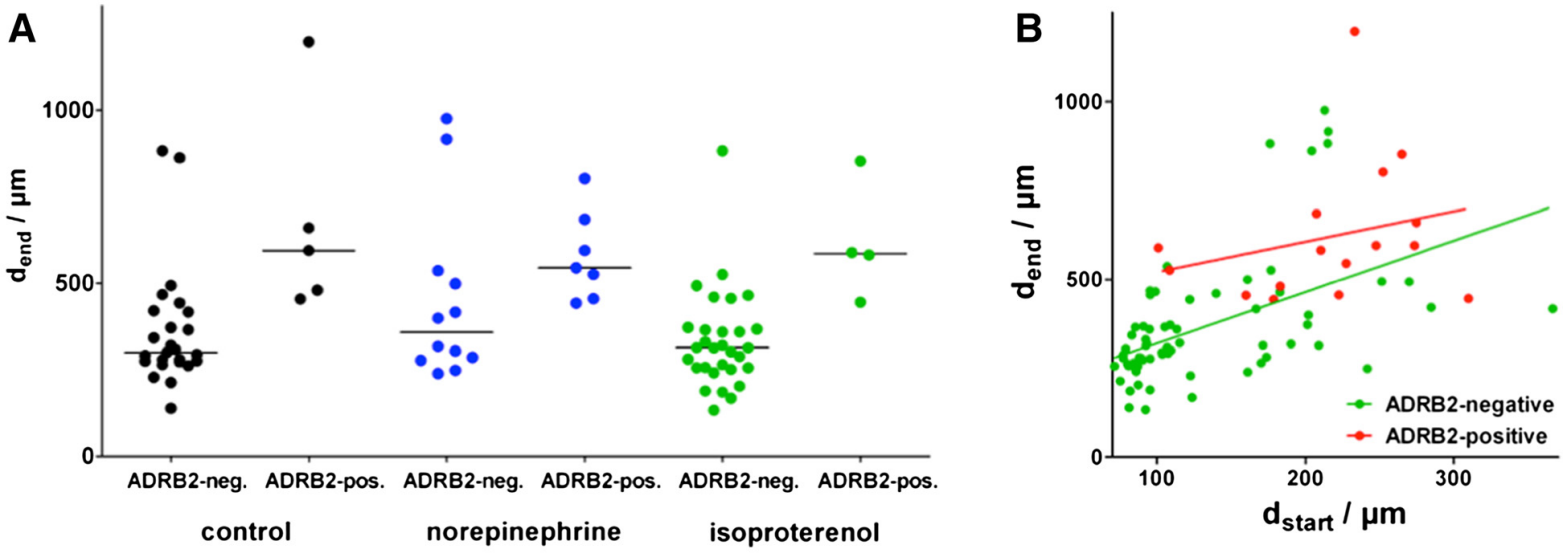
**Dependency of follicle size and growth on ADRB-2 presence.** Adrenergic stimulation does not affect follicular growth **(A)**. Secondary follicles isolated from macaque ovaries (n = 4 animals) were cultured in media containing FSH alone, or with norepinephrine (NE), isoproterenol (ISO). Follicles were grouped depending upon their diameter (at day 35) and the absence or presence of ADRB-2. Note that ADRB-positive follicles were significantly (P < 0.05) larger at d35 for all treatment groups. However, adrenergic treatment did not affect the diameters either ADRB-2-negative or -positive follicles (nonparametric one-way ANOVA (Kruskal-Wallis test) followed by Dunns post-test (alpha = 0.1)). Lines represent medians for the respective groups. **(B)** Follicle diameter and growth in culture depends on the presence of ADRB-2. Diameters of ADRB-2-positive (red; n = 16) and ADRB-2-negative follicles (green; n = 67) at beginning (d_start_) and end (d_end_) of culture. The lines show the linear correlation between diameters at the beginning and end of culture.

When the sizes of the surviving follicles were analyzed, those expressing ADRB-2 were significantly larger than ADRB-2 negative follicles (Figure 4A). However, adrenergic stimulation of ADRB-2-negative and -positive follicles, did not significantly affect follicular diameter at the end of the experiment (Figure 4A), nor its increase during in culture (data not shown; for both nonparametric one-way ANOVA (Kruskal-Wallis test) followed by Dunn’s post-test (alpha = 0.1)). Therefore, we pooled our data into two groups based on RT-PCR analysis of ADRB-2 in follicles at the end of culture: (1) ADRB-2-positive (n = 16) and ADRB-2-negative (n = 67) follicles. When analyzing changes of follicle size during culture, we found that ADRB-2-positive follicles had a significantly larger diameter, both at the beginning (d2) and the end (d35) of the culture experiment (Figure 4B; Mann–Whitney test, P < 0.0001 for both ages). There is also a general correlation of diameter at d2 and d35 evident (Figure 4B; correlation analysis, Spearman r = 0.6712, P < 0.0001). ADRB-2-positive follicles show a significantly larger increase in absolute follicular diameter (median = 360 μm, 95% CI interval: 302–505; n = 16) compared to their receptor negative counterparts (192 μm; 95% CI interval: 195–272; n = 67; data not shown; Mann–Whitney test, P < 0.0001). In contrast, we observed no difference in relative increase (data not shown; Mann–Whitney test, P = 0.85) due to the already larger diameters at the start of the experiments.

## Discussion

Our studies on the human ovary and that on a clinically relevant animal model, the rhesus monkey, gave identical results with regard to the expression pattern of ADRB-2. Several distinct cell types of the human and monkey ovary express ADRB-2 mRNA and protein, implying that they are potential targets for the neurotransmitter NE, which is derived primarily from ovarian innervation and was readily found in FF of large antral follicles. Based on this result and on the observed ADRB-2 expression by follicular GCs, 3D cultures of monkey preantral follicles were performed. Although the addition of ISO or NE did not promote growth of secondary macaque follicles during culture, mRNA expression of ADRB-2 was associated with the largest follicles at the time of isolation from the ovary and at the end of culture. This suggests ADRB-2-mediated actions in the development of follicles and that ADRB-2 expression may be a signature of fast growing follicles.

Both ADRB-2 protein and/or mRNA were localized to several ovarian compartments. Ovarian surface epithelium and interstitial cells, as well as the somatic cells of the follicles, TCs and GCs, express this receptor. The expression of ADRB-2 by the ovarian surface epithelium has to our knowledge not yet been reported. TCs, GCs and interstitial cells of several non-primate species are known to express ADRB-2 [10,11,24,36-39].

To what extend follicles in human or monkey ovary are innervated or associated with neuronal cellular elements, is not fully known ([1-7], yet GCs from primordial and primary follicles and TCs of larger follicles could in principle be in direct contact with nerve fibers [40] and could therefore be immediate targets for innervation-derived NE. Access of NE to GCs of larger follicles always depends on diffusion. In larger follicles, diffusion through the basal lamina and GC layers must occur and we found evidence that this indeed occurs during follicular growth. Thus, in FF of large monkey follicles, NE accumulated and reached levels that are much higher than serum levels. This is comparable to the situation in the human ovary [3]. The reason and mechanisms for accumulation in the FF remain unknown, but may be related to the expression of NE- and DA-transporters and storage-capacities of GCs with respect to catecholamines [3,4,41] and to the ability of oocytes to metabolize DA to NE [42]. These results establish that a ligand (NE in FF) and its receptor (ADRB-2 in GCs) co-exist in the large antral monkey follicle.

Immunoreactivity for ADBR-2 of GCs of small primordial and primary follicles was among the strongest observed in the ovary. In previous rat studies, in which postnatal ovaries were cultured, primordial and primary follicles, responded to ISO and NE, which enhanced growth of the follicles and the expression of FSHR [8]. The staining pattern suggests that a comparable role is possible in the human and monkey and may depend on the proximity to a source of NE, e.g. ovarian nerve fibers releasing NE. A growth and differentiation-promoting effect exerted by ADBR-2 stimulation is also supported by data from studies in stem cells, other organs [13,14] and tumor cells (see introduction e.g. [15]).

In our present study due to technical limitations, however, neither strongly immunopositive small follicles, nor large antral follicles could not be examined and only multilayered preantral monkey follicles, which showed weaker and heterogeneous ADRB-2-staining of GCs, could be cultured. It is possible that their patchy ADRB-2 expression is related to the fact that in our culture studies ISO and NE did not enhance follicular growth. This point clearly requires additional studies. Furthermore, in contrast to the study in neonatal rat ovary [8], monkey follicles in 3D culture depended on the presence of FSH in the medium ([31] and pilot studies). Thus it is possible that FSH, i.e. the prototype growth-promoting hormone for GCs, may have masked the actions of NE.

Despite these limitations, the analysis of the cultured follicles at the time of their harvest revealed that all surviving follicles examined, expressed FSHR and aromatase. Yet, of these growing follicles only a fraction co-expressed ADRB-2 at the mRNA level. Expression of ADRB-2 was not correlated with the treatment in culture but was significantly positively correlated with the size of the follicles and their ability to grow in culture.

It is therefore possible that expression of ADRB-2 is of advantage and that in vivo-growth of follicles, prior to isolation, may have already been affected by intra-ovarian NE. Thus, when the follicles expressing ADRB-2 were further analyzed and traced, it became clear that they were already larger at the time of isolation than the ones without ADRB-2. Unfortunately, their ADRB-2- as well as FSHR-status at time of isolation could not be verified. Yet, immunohistochemistry revealed that the patchy expression of ADRB-2 by GCs in small, multilayered follicles is replaced by a more homogeneous pattern in antral follicles. Given that NE, like in rat stimulates expression of FSHR [8], this may link actions, of NE with the observation that FSHR expression increases in growing follicles [43]. Thus it is possible that NE via ADRB-2 may stimulate both growth of small follicle and induce FSHR, and thus contribute to follicular development. Such an involvement in follicular development of primates, possibly in the initial growth as well as later in the large antral follicle, where NE is present in significant levels in the FF, requires further elucidation.

Finally, an intriguing possibility remains to be studied, namely whether ovarian ADRB-2 possess ligand-independent activity. This was reported in other systems and was located to certain parts of the receptor molecule [44]. If so, the mere presence of ADRB-2 may indicate spontaneous activity, which could occur in the absence of a ligand in many ovarian cells.

## Conclusions

Based on ADRB-2 expression, many potential targets for NE exist in the primate ovary, including monkey and human GCs. While access of NE to these targets may limit activation of ADRB-2, ligand-independent activity of ADRB-2 may occur and ADRB-2 expression is associated with the best-growing follicles in culture. This topic may be also of clinical interest. Thus drugs interfering with ADRB-2 are commonly used to treat high blood pressure and heart conditions. Based on the results of our study they may have ovarian targets in women. They may have actions in the human ovary and may possibly impact human fertility [28].

## References

[1] Lara HE, Porcile A, Espinoza J, Romero C, Luza SM, Fuhrer J (2001). Release of norepinephrine from human ovary: coupling to steroidogenic response. Endocrine.

[2] Greiner M, Paredes A, Araya V, Lara HE (2005). Role of stress and sympathetic innervation in the development of polycystic ovary syndrome. Endocrine.

[3] Saller S, Merz-Lange J, Raffael S, Hecht S, Pavlik R, Thaler C (2012). Norepinephrine, active norepinephrine transporter, and norepinephrine-metabolism are involved in the generation of reactive oxygen species in human ovarian granulosa cells. Endocrinol.

[4] Saller S, Kunz L, Berg D, Berg U, Lara H, Urra J (2014). Dopamine in human follicular fluid is associated with cellular uptake and metabolism-dependent generation of reactive oxygen species in granulosa cells: implications for physiology and pathology. Hum Reprod.

[5] D’Albora H, Anesetti G, Lombide P, Dees WL, Ojeda SR (2002). Intrinsic neurons in the mammalian ovary. Microsc Res Tech.

[6] Dees WL, Hiney JK, Schultea TD, Mayerhofer A, Danilchik M, Dissen GA (1995). The primate ovary contains a population of catecholaminergic neuron-like cells expressing nerve growth factor receptors. Endocrinol.

[7] Mayerhofer A, Smith GD, Danilchik M, Levine JE, Wolf DP, Dissen GA (1998). Oocytes are a source of catecholamines in the primate ovary: evidence for a cell-cell regulatory loop. Proc Natl Acad Sci U S A.

[8] Mayerhofer A, Dissen GA, Costa ME, Ojeda SR (1997). A role for neurotransmitters in early follicular development: induction of functional follicle-stimulating hormone receptors in newly formed follicles of the rat ovary. Endocrinol.

[9] Fohr KJ, Mayerhofer A, Sterzik K, Rudolf M, Rosenbusch B, Gratzl M (1993). Concerted action of human chorionic gonadotropin and norepinephrine on intracellular-free calcium in human granulosa-lutein cells: evidence for the presence of a functional alpha-adrenergic receptor. J Clin Endocrinol Metab.

[10] Itoh MT, Ishizuka B (2005). alpha1-Adrenergic receptor in rat ovary: presence and localization. Mol Cell Endocrinol.

[11] Morley P, Calaresu FR, Armstrong DT (1990). Catecholamines inhibit steroidogenesis by cultured porcine thecal cells. FEBS Lett.

[12] Aguado LI, Petrovic SL, Ojeda SR (1982). Ovarian beta-adrenergic receptors during the onset of puberty: characterization, distribution, and coupling to steroidogenic responses. Endocrinol.

[13] Yeh CK, Chandrasekar B, Lin AL, Dang H, Kamat A, Zhu B (2012). Cellular signals underlying beta-adrenergic receptor mediated salivary gland enlargement. Differ; Res Biol Divers.

[14] Yan L, Jia Z, Cui J, Yang H, Yang H, Zhang Y (2011). Beta-adrenergic signals regulate cardiac differentiation of mouse embryonic stem cells via mitogen-activated protein kinase pathways. Dev Growth Differ.

[15] Tang J, Li Z, Lu L, Cho CH (2013). beta-Adrenergic system, a backstage manipulator regulating tumour progression and drug target in cancer therapy. Semin Cancer Biol.

[16] Armaiz-Pena GN, Allen JK, Cruz A, Stone RL, Nick AM, Lin YG (2013). Src activation by beta-adrenoreceptors is a key switch for tumour metastasis. Nat Commun.

[17] Moretti S, Massi D, Farini V, Baroni G, Parri M, Innocenti S (2013). beta-adrenoceptors are upregulated in human melanoma and their activation releases pro-tumorigenic cytokines and metalloproteases in melanoma cell lines. Lab Investi; JTech Methods and Pathol.

[18] Cole SW, Sood AK (2012). Molecular pathways: beta-adrenergic signaling in cancer. Clin Cancer Res: an official J American Assoc for Cancer Res.

[19] Luna SL, Neuman S, Aguilera J, Brown DI, Lara HE (2012). In vivo beta-adrenergic blockade by propranolol prevents isoproterenol-induced polycystic ovary in adult rats. Horm Metab Res = Hormon- und Stoffwechselforschung = Hormones et metabolisme.

[20] Sam S, Dunaif A (2003). Polycystic ovary syndrome: syndrome XX?. Trends Endocrinol Metab: TEM.

[21] Ehrmann DA (2005). Polycystic ovary syndrome. N Engl J Med.

[22] Heider U, Pedal I, Spanel-Borowski K (2001). Increase in nerve fibers and loss of mast cells in polycystic and postmenopausal ovaries. Fertil Steril.

[23] Stener-Victorin E, Jedel E, Manneras L (2008). Acupuncture in polycystic ovary syndrome: current experimental and clinical evidence. J Neuroendocrinol.

[24] Manni L, Cajander S, Lundeberg T, Naylor AS, Aloe L, Holmang A (2005). Effect of exercise on ovarian morphology and expression of nerve growth factor and alpha (1)- and beta (2)-adrenergic receptors in rats with steroid-induced polycystic ovaries. J Neuroendocrinol.

[25] Manni L, Holmang A, Lundeberg T, Aloe L, Stener-Victorin E (2005). Ovarian expression of alpha (1)- and beta (2)-adrenoceptors and p75 neurotrophin receptors in rats with steroid-induced polycystic ovaries. Auton Neurosci: Basic Clin.

[26] Dissen GA, Garcia-Rudaz C, Paredes A, Mayer C, Mayerhofer A, Ojeda SR (2009). Excessive ovarian production of nerve growth factor facilitates development of cystic ovarian morphology in mice and is a feature of polycystic ovarian syndrome in humans. Endocrinol.

[27] Lara HE, Ferruz JL, Luza S, Bustamante DA, Borges Y, Ojeda SR (1993). Activation of ovarian sympathetic nerves in polycystic ovary syndrome. Endocrinol.

[28] Frishman WH (2003). Cardiology patient page. Beta-adrenergic blockers. Circulation.

[29] Mayerhofer A, Kunz L, Krieger A, Proskocil B, Spindel E, Amsterdam A (2006). FSH regulates acetycholine production by ovarian granulosa cells. Reprod Biol Endocrinol: RB&E.

[30] Kampfer C, Saller S, Windschuttl S, Berg D, Berg U, Mayerhofer A (2014). Pigment-epithelium derived factor (PEDF) and the human ovary: a role in the generation of ROS in granulosa cells. Life Sci.

[31] Xu M, West-Farrell ER, Stouffer RL, Shea LD, Woodruff TK, Zelinski MB (2009). Encapsulated three-dimensional culture supports development of nonhuman primate secondary follicles. Biol Reprod.

[32] Xu J, Lawson MS, Yeoman RR, Molskness TA, Ting AY, Stouffer RL (2013). Fibrin promotes development and function of macaque primary follicles during encapsulated three-dimensional culture. Hum Reprod.

[33] Xu J, Xu M, Bernuci MP, Fisher TE, Shea LD, Woodruff TK (2013). Primate follicular development and oocyte maturation in vitro. Adv Exp Med Biol.

[34] Adam M, Schwarzer JU, Kohn FM, Strauss L, Poutanen M, Mayerhofer A (2011). Mast cell tryptase stimulates production of decorin by human testicular peritubular cells: possible role of decorin in male infertility by interfering with growth factor signaling. Hum Reprod.

[35] Wolf DP, Vandevoort CA, Meyer-Haas GR, Zelinski-Wooten MB, Hess DL, Baughman WL (1989). In vitro fertilization and embryo transfer in the rhesus monkey. Biol Reprod.

[36] Perkins SN, Cronin MJ, Veldhuis JD (1986). Properties of beta-adrenergic receptors on porcine corpora lutea and granulosa cells. Endocrinol.

[37] Kannisto P, Owman C, Walles B (1985). Involvement of local adrenergic receptors in the process of ovulation in gonadotrophin-primed immature rats. J Reprod Fertil.

[38] Laszlovszky I, Erdo SL (1983). Characterization of beta-adrenergic receptors in rat ovary. Eur J Pharmacol.

[39] Luck MR, Munker M (1991). Beta adrenoceptors mediate the catecholamine-induced stimulation of oxytocin secretion from cultured bovine granulosa cells. Reprod Fertil Dev.

[40] Hernandez ER, Jimenez JL, Payne DW, Adashi EY (1988). Adrenergic regulation of ovarian androgen biosynthesis is mediated via beta 2-adrenergic theca-interstitial cell recognition sites. Endocrinol.

[41] Greiner M, Paredes A, Rey-Ares V, Saller S, Mayerhofer A, Lara HE (2008). Catecholamine uptake, storage, and regulated release by ovarian granulosa cells. Endocrinol.

[42] Mayerhofer A, Danilchik M, Pau KY, Lara HE, Russell LD, Ojeda SR (1996). Testis of prepubertal rhesus monkeys receives a dual catecholaminergic input provided by the extrinsic innervation and an intragonadal source of catecholamines. Biol Reprod.

[43] Weinbauer GF, Niehoff M, Niehaus M, Srivastav S, Fuchs A, Van Esch E (2008). Physiology and endocrinology of the ovarian cycle in Macaques. Toxicol Pathol.

[44] Chakir K, Xiang Y, Yang D, Zhang SJ, Cheng H, Kobilka BK (2003). The third intracellular loop and the carboxyl terminus of beta2-adrenergic receptor confer spontaneous activity of the receptor. Mol Pharmacol.

